# Midgut bacterial diversity of a leaf-mining beetle, *Dactylispa xanthospila* (Gestro) (Coleoptera: Chrysomelidae: Cassidinae)

**DOI:** 10.3897/BDJ.9.e62843

**Published:** 2021-05-10

**Authors:** Lixing Cui, Qingyun Guo, Xuexiong Wang, Kevin Jan Duffy, Xiaohua Dai

**Affiliations:** 1 Leafminer Group, School of Life Sciences, Gannan Normal University, Ganzhou, China Leafminer Group, School of Life Sciences, Gannan Normal University Ganzhou China; 2 Institute of Systems Science, Durban University of Technology, Durban, South Africa Institute of Systems Science, Durban University of Technology Durban South Africa; 3 National Navel-Orange Engineering Research Center, Ganzhou, China National Navel-Orange Engineering Research Center Ganzhou China

**Keywords:** gut microbita, gut microbiome, 16S rRNA, metabolic pathway analysis

## Abstract

Microorganisms play an essential role in the growth and development of numerous insect species. In this study, the total DNA from the midgut of adults of *Dactylispa xanthospila* were isolated and bacterial 16S rRNA sequenced using the high-throughput Illumina MiSeq platform. Then, the composition and diversity of the midgut bacterial community were analysed with QIIME2. The results showed the midgut bacteria of *D. xanthospila* belong to 30 phyla, 64 classes, 135 orders, 207 families and 369 genera. At the phylum level, Proteobacteria, Bacteroidetes and Firmicutes were the dominant bacteria, accounting for 91.95%, 3.44% and 2.53%, respectively. The top five families are Enterobacteriaceae (69.51%), Caulobacteraceae (5.24%), Rhizobiaceae (4.61%), Sphingomonadaceae (4.23%) and Comamonadaceae (2.67%). The bacterial community's primary functions are carbohydrate metabolism, amino acid metabolism and cofactor and vitamin metabolism, which are important for the nutritional requirements of plant-feeding insects.

## Introduction

As special internal environments, animal guts host abundant microorganisms and the gut microbiome is one of the essential parts of the animal-microbe super-organism (Kramer and Bressan 2015, Salvucci 2019, Sleator 2010). After long-term co-evolution, gut microbes and animal hosts have shaped different kinds of ecological relationships, including commensalism, mutualism and parasitism (Backhed 2005, Hooper and Gordon 2001). Insects comprise numerous species, have various habitats and use diverse foods (Basset et al. 2012, Shi et al. 2010) and thus have correspondingly evolved diverse gut characteristics and microbiota (Engel and Moran 2013). Some gut microorganisms improve the host’s nutritional, digestive and reproductive fitness and even pathogen defence (Chen et al. 2020, Douglas 2015, Engel and Moran 2013, Liu et al. 2020, Wang et al. 2020). Gut microbial composition of insects are largely affected by host feeding habits (Colman et al. 2012, Wang et al. 2020, Liu et al. 2020, Yun et al. 2014). Moreover, gut microbiota can vary when insects feed on different plant parts of the same host species (Chen et al. 2020).

Many insects have associated microbial symbionts in their midgut that provide ecologically-important benefits to the host. Many of these bacteria can improve the host's health or life span (Douglas 2015, Behar et al. 2008b). They are indispensable for the normal growth and development of host insects. Therefore, the relationship between microorganisms and hosts in insects has gradually become one of the hotspots in entomological research. Besides, the extensive application of various biological techniques in entomology and microbiology has promoted the research on the co-evolution of gut microorganisms and host insects (Wang et al. 2020).

*Dactylispa xanthospila* (Gestro) (Coleoptera: Chrysomelidae: Cassidinae) is mainly distributed in the Oriental Region. In China, they are is mainly found in East China, South China and southwest China (Chen et al. 1986). *Dactylispa xanthospila* is a leaf-miner feeding inside the leaves of several weeds of Poaceae. In this paper, the midgut bacterial 16S rRNA of *D. xanthospila* adults were high-throughput sequenced and the composition and diversity of the midgut bacterial community were analysed.

## Materials and methods

### Sample collection

*Dactylispa xanthospila* adults and larvae were collected on *Pogonatherum crinitum* (Thunb.) Kunth and *Arthraxon prionodes* (Steud.) Dandy (Poaceae) at Damingshan, Nanning, China (23.52 N, 108.49 E) on 15 August 2019. Voucher specimens of the beetles were deposited in Nanling Herbarium, Gannan Normal University (GNNU). The larvae were reared to adults in the lab.

After treatment by 48 h of starvation to evacate food plant materials (Vilanova et al. 2016), *D. xanthospila* adults were soaked in 70% ethyl alcohol for 3 min and then washed three times with sterile deionised water to remove exogenous contaminants. The guts were then dissected under sterile deionised water, using sterilised tweezers and eye scissors under aseptic conditions. There were three replicates and each replicate consisted of a mixture of samples from 10 adults. A total of 30 adults were dissected. The gut was immediately frozen at -80°C for subsequent DNA extraction.

### DNA extraction and sequencing

Extractions of DNA from the gut samples were performed using a Mag-Bind Soil DNA extraction kit (Omega, Norcross, GA, USA) according to the manufacturer’s instructions. The V3-V4 hypervariable region of the 16S rRNA gene was amplified with the universal primers 338F (5’-ACTCCTACGGGAGGCAGCA-3’) and 806R (5’-GGACTACHVGGGTWTCTAAT-3’) (Mizrahi-Man et al. 2013). The amplification reactions were carried out in a 25 μl volume, containing 5 μl of Q5 reaction buffer (5×), 5 μl of Q5 High-Fidelity GC buffer (5×), 2 μl of dNTPs (2.5 mM), 1 μl each of forward and reverse primer (10 uM), 2 μl of DNA template, 0.25 μl of Q5 High-Fidelity DNA Polymerase (5 U/μl) and made up to 25 μl with sterile H_2_O. The fragments were amplified under the following conditions: denaturation at 98°C for 2 min, followed by 27 cycles of denaturation at 98°C for 15 s, annealing at 55°C for 30 s and extension at 72°C for 30 s, with a final extension at 72°C for 5 min. The PCR products were purified with magnetic beads (Vazyme VAHTSTM DNA Clean Beads). The purified PCR products were quantified with the fluorescent reagent (Quant-iT PicoGreen dsDNA Assay Kit0, Life, USA), using a Microplate reader (FLx800, BioTek, USA). The sequencing library was prepared with TruSeq Nano DNA LT Library Prep Kit, Illumina (USA) through mixing each sample in a corresponding proportion according to the requirements of the sequencing quantity of each sample and the fluorescence quantification results. Then, all PCR products were sequenced on an Illumina Miseq platform using 2 × 300 base pairs (bp) paired-end reads (Personalbio, Shanghai, China). The reference number of sequence data is MbPL201910038.

### Data analysis

Microbiome bioinformatics was performed with QIIME 2 2019.4 (Bolyen et al. 2019) with slight modification according to the official tutorials (https://docs.qiime2.org/2019.4/tutorials/). First, both unmatched sequences and primer fragments of matched sequences were removed with the cutadapt plugin (Martin 2011). Sequences were then quality filtered, denoised, merged and the chimera removed, using the DADA2 plugin (Callahan et al. 2016). The classification-sklearn algorithm of QIIME2 (Nicholas et al. 2018) was used to annotate the representative sequences of each operational taxonomic unit (OTU) by a pre-trained Naive Bayes classifier, using the SILVA database (Release 138) (Quast et al. 2012) under default parameters. The phylogeny tree was constructed with FastTree (Price et al. 2010). The taxonomic composition of midgut bacteria at different taxonomic levels was visually presented with the Krona software (Ondov et al. 2011). All data used in bacterial abundance analyses are available in Suppl. material 1.

### Metabolic pathway analysis of midgut bacteria

The abundance of marker gene sequences was analysed with PICRUSt2 software to predict the functional abundance of different samples (Douglas et al. 2020). First, the 16S rRNA gene sequences of the sequenced *D. xanthospila* were aligned, the evolutionary tree was constructed and the gene functional profiles of their common ancestors were inferred. A new evolutionary tree was then constructed by aligning 16S rRNA feature sequences with reference sequences. Using the R package ‘castor’ (Louca and Doebeli 2018), the nearest sequence species of the characteristic sequence were inferred according to the copy number of the gene family corresponding to the reference sequence in the evolutionary tree and then the copy number of the gene family was obtained. When the nearest sequence species index (NTSI) of each sequence was available, sequences with NTSI > 2 were excluded from subsequent analysis. Combining the abundance of characteristic sequences of each sample, the copy number of gene families of each sample was calculated. Finally, the gene family was "mapped" to MetaCyc (https://metacyc.org/) (Caspi et al. 2006) and KEGG (https://www.kegg.jp/) (Kanehisa and Goto 2000). In the database, MinPath was used to infer the existence of metabolic pathways and then the abundance data of these metabolic pathways in each sample were obtained (Ye and Doak 2009).

## Results and discussion

**Taxonomic composition of midgut bacteria**

**Figure 1.**
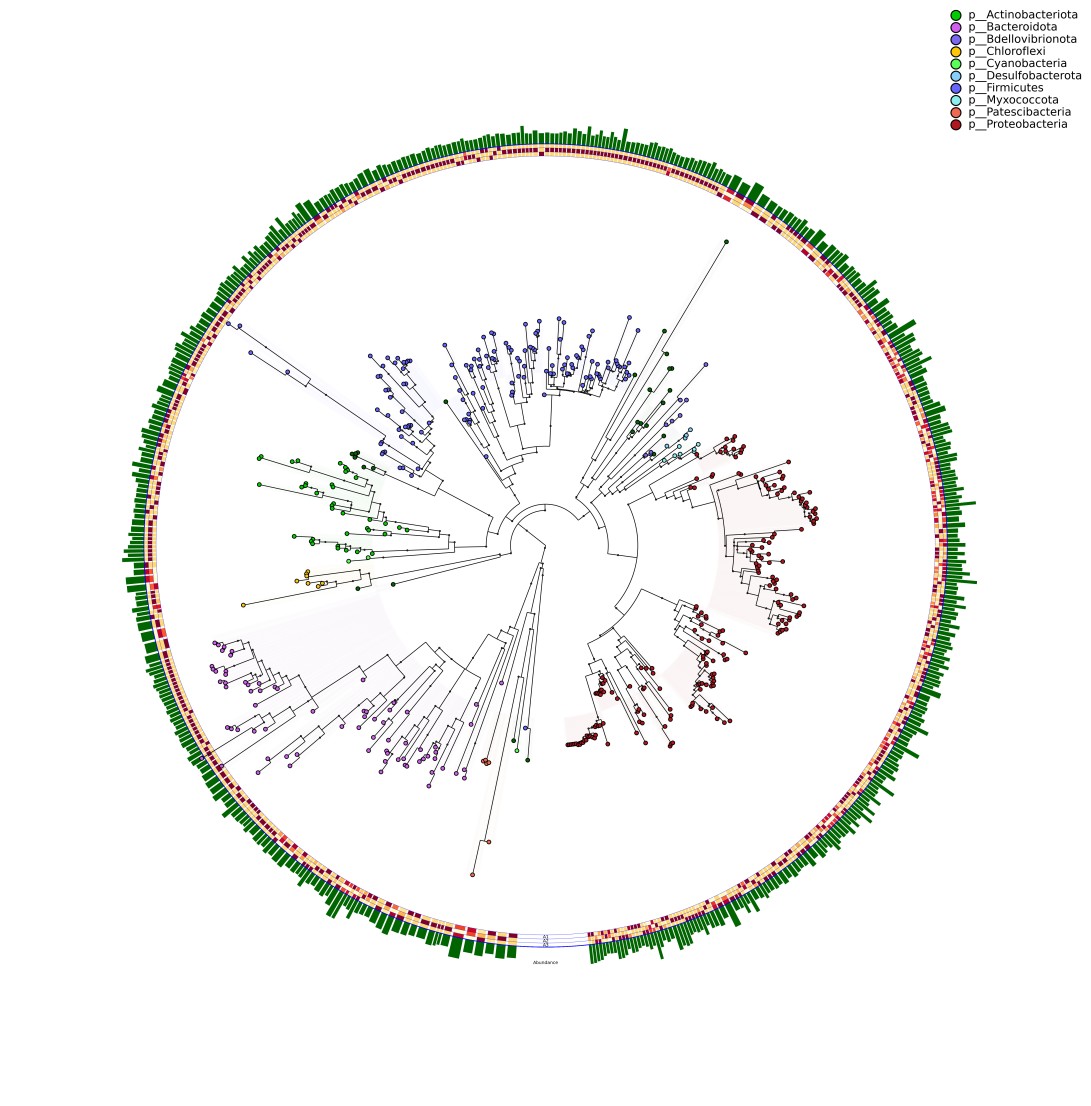
Graphlan evolutionary tree diagram of *D. xanthospila (Gestro)* midgut bacteria.

According to the OTU classification, the midgut bacteria of *D. xanthospila* belong to 30 phyla, 64 classes, 135 orders, 207 families and 369 genera (Suppl. material 1). From the phylogenetic tree, it can be seen that three bacteria phyla (Proteobacteria, Bacteroidetes and Firmicutes) have much richer species than any other phyla in the midgut of *D. xanthospila* (Fig. 1).

**Figure 2.**
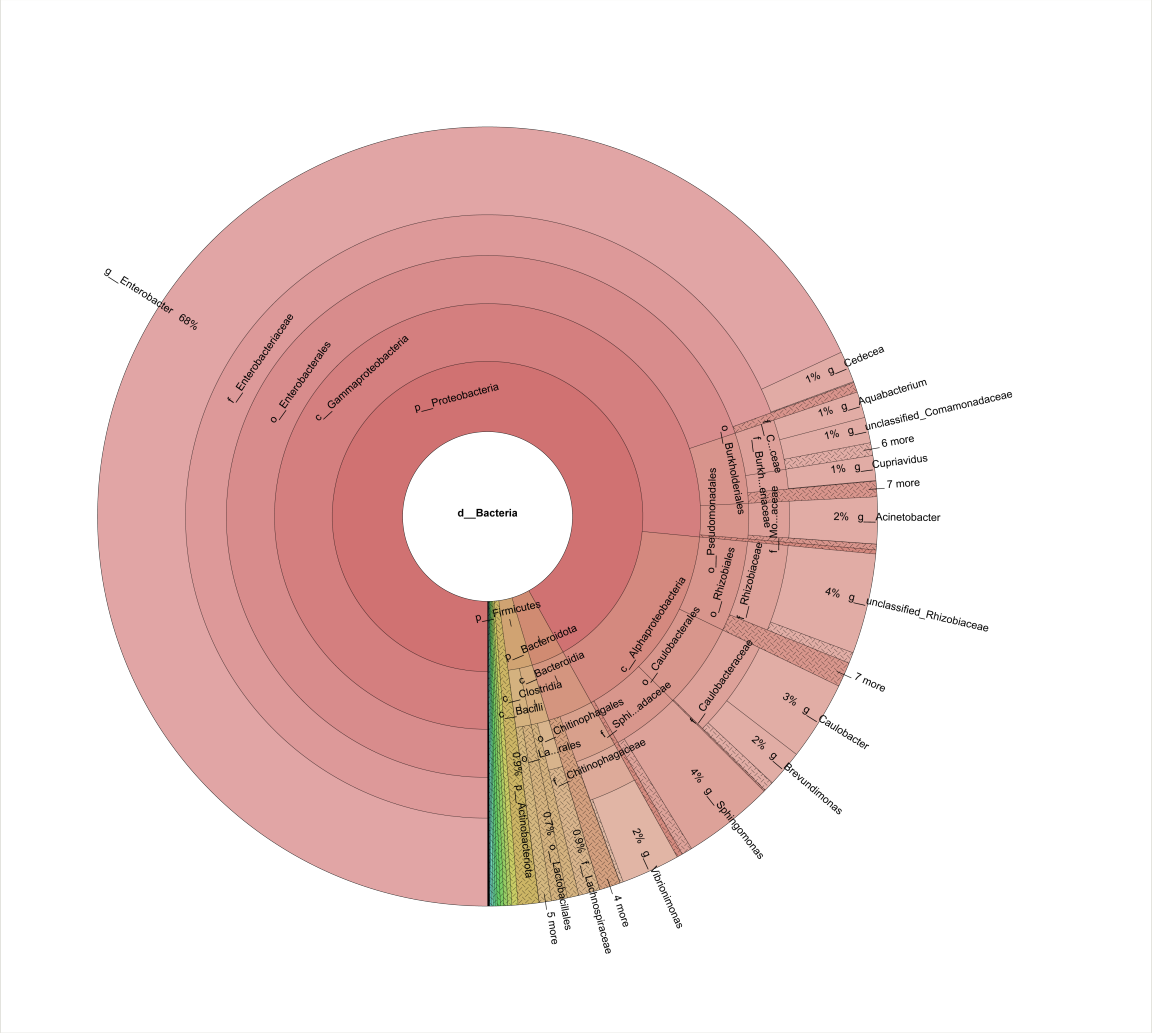
The composition of *D. xanthospila* midgut bacteria at different taxonomic levels. The letter before each scientific name stands for the corresponding taxonomic level: d - domain; p - phylum; c - class; o - order; f - family; g - genus.

At the phylum taxonomic level, the midgut bacteria of *D. xanthospila* belong to 30 phyla (Suppl. material 1). Proteobacteria, Bacteroidetes and Firmicutes were the dominant bacteria, accounting for 91.95%, 3.44% and 2.53%, respectively (Fig. 2;Suppl. material 1). Gut microbiota of 218 insect species in 21 orders are generally dominated by Firmicutes (62.1%) and Proteobacteria (20.7%) (Yun et al. 2014). For example, Proteobacteria and Firmicutes are amongst the dominant flora in the intestinal tract of lepidopteran insects, such as *Plutella xylostella*, *Lymantria dispar*, *Helicoverpa armigera, Spodoptera littoralis, Ectropis obliqua, E. grisescens* and *Bombyx Mori* （Rajan et al. 2020, Shinde et al. 2019, Xia et al. 2017, Zeng et al. 2019, Chen et al. 2016, Wang et al. 2020). Proteobacteria and Firmicutes are also dominant in the intestinal bacterial community of other insects, such as *Bactrocera tau*, *Ceratitis capitata*, *Procecidochares utilis* and *Lutzomyia longipalpis* of Diptera, *Anophora glabripennis* of Coleoptera and *Schistocerca gregaria* of Orthoptera (Behar et al. 2008b, Dillon et al. 2010, Gouveia et al. 2008, Prabhakar et al. 2013, Schloss et al. 2006). Bacteroidetes dominates in the gut microbiota of some insect species (Tagliavia et al. 2014, Yun et al. 2014, Ferguson et al. 2018). However, the order of dominant bacteria phyla in insect guts differs amongst different host species (He et al. 2001, Huang and Zhang 2017, Wang et al. 2011, Xia et al. 2013).

At the class taxonomic level, the midgut bacteria of *D. xanthospila* belong to 64 classes, including Gammaproteobacteria, Alphaproteobacteria, Bacteroidia and Clostridia (Suppl. material 1). Gammaproteobacteria was the dominant class, accounting for 76.53%, followed by Alphaproteobacteria and Bacteroidia, with an abundance of 15.38% and 3.44%, respectively (Fig. 2;Suppl. material 1). Gammaproteobacteria generally dominates in insect guts, with a great impact on insects growth and development (Karamipour et al. 2016, Kashkouli et al. 2019). Gammaproteobacteria in the intestinal tract of bees can encode pectin-degrading enzymes and degrade pectin in pollen, indicating that Gammaproteobacteria can help bees digest food (Engel et al. 2012). The biological functions of Alphaproteobacteria on the host mainly include reproductive regulation, life history suitability and tolerance to the external environment (Zhang et al. 2017). Bacteroidia are dominant in almost all gut microbiome of dictyopteran insects; however, cockroaches and termites share fewer Bacteroidia species than expected (Sabree and Moran 2014). Clostridia are also abundant in dictyopteran hosts including mantids, cockroaches and termites (Sabree and Moran 2014).

At the order taxonomic level, the midgut bacteria of *D. xanthospila* are distributed in 135 orders, including Enterobacteriales, Rhizobiales, Caulobacterales, Burkholderiales and Sphingomonadales (Suppl. material 1). The abundance of Enterobacteriales, Rhizobiales and Caulobacterales was 69.83%, 5.63% and 5.29%, respectively and other orders accounted for 19.25% (Fig. 2;Suppl. material 1). Enterobacteriales is one dominant order in the gut biota of *Spodoptera litura*, reared either on taro leaves or on artificial diet (Xia et al. 2020). The proportion of Enterobacteriales is about 45% amongst the midgut bacterial orders of diamondback moth (*Plutella xylostella*) (Xia et al. 2013, Xia et al. 2017). The wide distribution of Rhizobiales in ants is connected with their herbivory adaptations; Rhizobiales could increase the nitrogen supplies for plant-feeding ants (Russell et al. 2009). Enterobacteriales is the most abundant gut bacterial order in the blood-suking bugs (*Triatoma dimidiata*) found on porcupine, while Burkholderiales for those live on dogs (Dumonteil et al. 2018).

At the family taxonomic level, the midgut bacteria of *D. xanthospila* belong to 207 families (Suppl. material 1). Amongst them, five families with an abundance larger than 2% are Enterobacteriaceae (69.51%), Caulobacteraceae (5.24%), Rhizobiaceae (4.61%), Sphingomonadaceae (4.23%) and Comamonadaceae (2.67%) (Fig. 2;Suppl. material 1). Enterobacteriaceae was also dominant in the intestine of Tephritidae (Behar et al. 2008a, Capuzzo et al. 2005). Some Enterobacteriaceae can help the host degrade cellulose, xylan, pectin and other polysaccharide substances in the plant cell wall and promote the host's digestion of food (Abbott and Boraston 2008, Anand et al. 2010). Enterobacteriaceae is considered to be the most prevalent symbiotic microorganism in dipteran insects (Kuzina et al. 2001). Some Enterobacteriaceae play an important role in plastic biodegradation (Xu et al. 2020). Many Enterobacteriaceae can fix nitrogen and produce necessary nutrients for the hosts (Behar et al. 2005). Enterobacteriaceae can also produce anti-fungal compounds for insect resistance to many pathogenic fungi (Oh et al. 2015).

At the genus taxonomic level, 369 genera of bacteria were annotated in the midgut samples of *D. xanthospila* (Fig. 2;Suppl. material 1) . Amongst them, bacteria mainly belong to Enterobacter, one unclassified genus of Rhizobiaceae, Sphingomonas, Caulobacter, Vibrionimonas, Acinetobacter, Brevundimonas and Cedecea (Fig. 2;Suppl. material 1). Enterobacter is the dominant genus of bacteria, accounting for 68.07% of the total (Suppl. material 1). Sphingomonas can tolerate extreme nutrient deprivation (Fegatella and Cavicchioli 2000) and degrade complex organic matter (Gong et al. 2016). Some Sphingomonas species can also produce valuable biopolymers, such as beta-carotene and gellan gum (Silva et al. 2004, Wang et al. 2006). Sphingomonas could also protect the host plants against pathogens (Innerebner et al. 2011, Laskin and White 1999). However, some Sphingomonas species can cause infection to plant roots or animal wounds (Zhao et al. 2016). In a leaf-mining moth *Diaphania pyloalis*, *Wolbachia* can account for 40.60% of the total gut bacterial genera (Chen et al. 2018). However, no *Wolbachia* were detected in our leaf-mining beetle *D. xanthospila*.

**Metabolic pathways of midgut bacteria**

**Figure 3.**
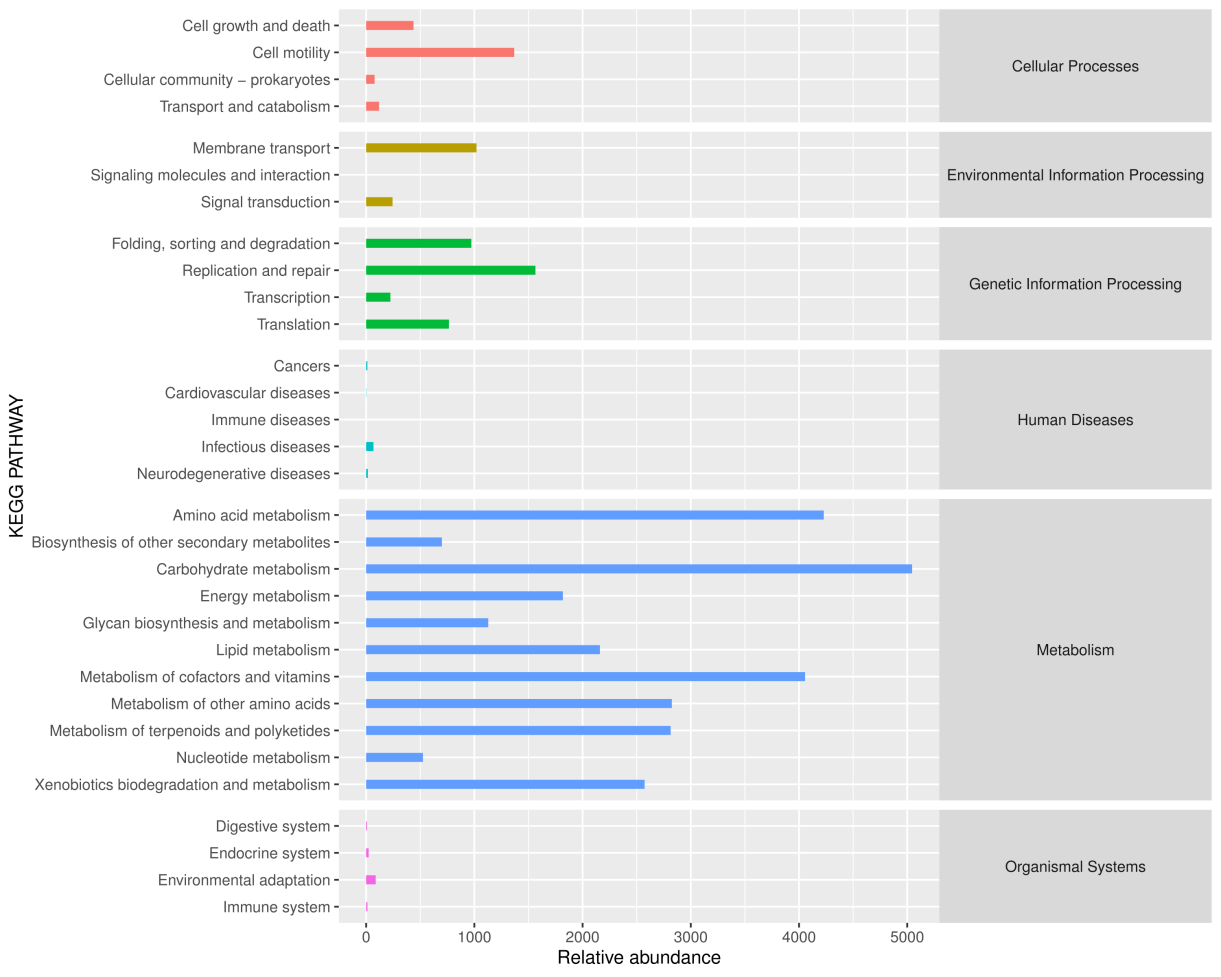
Abundance map of secondary functional pathways of *D. xanthospila* as predicted, based on the KEGG database. Note: The abscissa is the abundance of the functional pathways (in units of KO per million), the ordinate is the functional pathway at the second classification level of KEGG and the rightmost division is the first hierarchical pathway to which the pathway belongs. This figure shows the average abundance of all samples.

**Figure 4.**
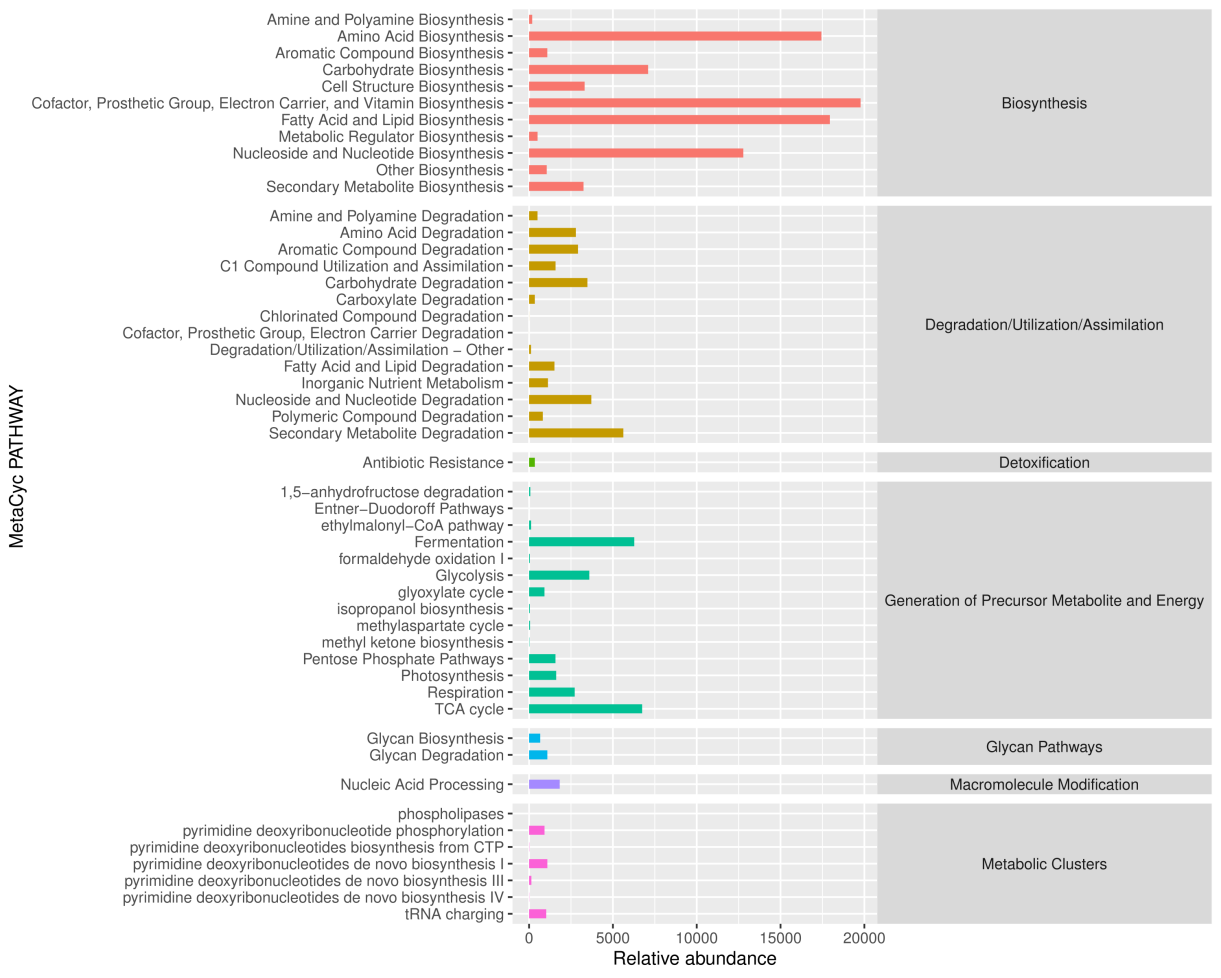
Abundance map of secondary functional pathways of *D. xanthospila* as predicted, based on the MetaCyc database. Note: The abscissa is the abundance of the functional pathway (in the unit of KO per million), the ordinate is the functional pathway at the second classification level of MetaCyc and the rightmost division is the first hierarchical pathway to which the pathway belongs. This figure shows the average abundance of all samples.

Primary functions of midgut bacteria in the *D. xanthospila* adults are metabolism and biosynthesis (Figs 3, 4). There are several important metabolic pathways, such as carbohydrate metabolism, amino acid metabolism and metabolism of cofactors and vitamins (Fig. 3). The primary biosynthetic pathways are (1) cofactor, prosthetic group, electron carrier and vitamin biosynthesis; (2) amino acid biosynthesis; (3) fatty acid and lipid biosynthesis; (4) nucleoside and nucleotide biosynthesis (Fig. 4).

Many herbivorous insects can neither produce all the necessary vitamins and amino acids nor obtain them from the food plants (Behmer 2009, Skidmore and Hansen 2017). However, some gut bacteria can take part in the biosynthesis of amino acids, vitamins and cofactors to compensate for the nutrition shortage of plant-feeding (Dillon and Dillon 2004, Skidmore and Hansen 2017). For example, both *Pseudomonas* and *Acinetobacter* play very important roles in the nutrient supplements of willow galling sawflies (Michell and Nyman 2021). Diamondback moth (*Plutella xylostella*) could not synthesise histidine (His) and threonine (Thr) by itself, but there existed the complete synthesis pathways of the two amino acids in midgut microbiota (Xia et al. 2017). Symbiotic bacteria in a wood-feeding termite gut could help with lignocellulose degradation (Warnecke et al. 2007). Gut bacteria in honey bees could make vitamin B12 for the host (Engel and Moran 2013). In the glassy-winged sharpshooter (*Homalodisca coagulata*), the gammaproteobacterium *Baumannia cicadellinicola* produces vitamins and cofactors, while the Bacteroidetes species *Sulcia muelleri* synthesises essential amino acids (Wu et al. 2006). Gut microbes provide many necessary amino acids for their associated host - the Asian longhorned beetle (*Anoplophora glabripennis*) (Ayayee et al. 2015).

In the midgut microbiota of *D. xanthospila*, nearly two-thirds are plant-fermentation-related bacteria, such as Enterobacteriaceae and Brucellaceae. *D. xanthospila* is a herbivorous insect and these bacteria may help *D. xanthospila* with the digestion of plant tissues. However, few studies on the gut microbiota of different leaf-mining insect groups have been carried out. Therefore, whether there are any microbes which might be linked to the leaf-mining habits needs further verification.

## Supplementary Material

F0D17FA8-8CA1-57CD-87E0-AA119550FFF2 10.3897/BDJ.9.e62843.suppl1 Supplementary material 1 The composition of *D. xanthospila* midgut bacteria at different taxonomic levels Data type relative abundance File: oo_531971.xlsx https://binary.pensoft.net/file/531971 Lixing Cui, Xiaohua Dai

## References

[B7006636] Abbott D. Wade, Boraston Alisdair B. (2008). Structural Biology of Pectin Degradation by Enterobacteriaceae. Microbiology and Molecular Biology Reviews.

[B7006623] Anand A. Alwin Prem, Vennison S. John, Sankar S. Gowri, Prabhu D. Immanual Gilwax, Vasan P. Thirumalai, Raghuraman T., Geoffrey C. Jerome, Vendan S. Ezhil (2010). Isolation and Characterization of Bacteria from the Gut of Bombyx morithat Degrade Cellulose, Xylan, Pectin and Starch and Their Impact on Digestion. Journal of Insect Science.

[B6903027] Ayayee Paul A, Larsen Thomas, Rosa Cristina, Felton Gary W, Ferry James G, Hoover Kelli (2015). Essential Amino Acid Supplementation by Gut Microbes of a Wood-Feeding Cerambycid.. Environmental Entomology.

[B6424717] Backhed F. (2005). Host-bacterial mutualism in the human intestine. Science.

[B6423995] Basset Y., Cizek L., Cuenoud P., Didham R. K., Guilhaumon F., Missa O., Novotny V., Odegaard F., Roslin T., Schmidl J., Tishechkin A. K., Winchester N. N., Roubik D. W., Aberlenc H. P., Bail J., Barrios H., Bridle J. R., Castano-Meneses G., Corbara B., Curletti G., Duarte da Rocha W., De Bakker D., Delabie J. H., Dejean A., Fagan L. L., Floren A., Kitching R. L., Medianero E., Miller S. E., Gama de Oliveira E., Orivel J., Pollet M., Rapp M., Ribeiro S. P., Roisin Y., Schmidt J. B., Sorensen L., Leponce M. (2012). Arthropod diversity in a tropical forest. Science.

[B7006668] Behar A, Yuval B, Jurkevitch E (2005). Enterobacteria-mediated nitrogen fixation in natural populations of the fruit fly Ceratitis
capitata.. Molecular Ecology.

[B6424038] Behar A., Jurkevitch E., Yuval B. (2008). Bringing back the fruit into fruit fly–bacteria interactions. Molecular Ecology.

[B6423593] Behar A., Yuval B., Jurkevitch E. (2008). Gut bacterial communities in the Mediterranean fruit fly (*Ceratitis
capitata*) and their impact on host longevity. Journal of Insect Physiology.

[B6903018] Behmer Spencer T. (2009). Insect Herbivore Nutrient Regulation. Annual Review of Entomology.

[B6424065] Bolyen Evan, Rideout Jai Ram, Dillon Matthew R., Bokulich Nicholas A., Abnet Christian C., Al-Ghalith Gabriel A., Alexander Harriet, Alm Eric J., Arumugam Manimozhiyan, Asnicar Francesco, Bai Yang, Bisanz Jordan E., Bittinger Kyle, Brejnrod Asker, Brislawn Colin J., Brown C. Titus, Callahan Benjamin J., Caraballo-Rodríguez Andrés Mauricio, Chase John, Cope Emily K., SilvaChristian Diener Ricardo Da, Dorrestein Pieter C., Douglas Gavin M., Durall Daniel M., Duvallet Claire, Edwardson Christian F., Ernst Madeleine, Estaki Mehrbod, Fouquier Jennifer, Gauglitz Julia M., Gibbons Sean M., Gibson Deanna L., Gonzalez Antonio, Gorlick Kestrel, Guo Jiarong, Hillmann Benjamin, Holmes Susan, Holste Hannes, Huttenhower Curtis, Huttley Gavin A., Janssen Stefan, Jarmusch Alan K., Jiang Lingjing, Kaehler Benjamin D., Kang Kyo Bin, Keefe Christopher R., Keim Paul, Kelley Scott T., Knights Dan, Koester Irina, Kosciolek Tomasz, Kreps Jorden, Langille Morgan G. I., Lee Joslynn, Ley Ruth, Liu Yong-Xin, Loftfield Erikka, Lozupone Catherine, Maher Massoud, Marotz Clarisse, Martin Bryan D., McDonald Daniel, McIver Lauren J., Melnik Alexey V., Metcalf Jessica L., Morgan Sydney C., Morton Jamie T., Naimey Ahmad Turan, Navas-Molina Jose A., Nothias Louis Felix, Orchanian Stephanie B., Pearson Talima, Peoples Samuel L., Petras Daniel, Preuss Mary Lai, Pruesse Elmar, Rasmussen Lasse Buur, Rivers Adam, Robeson II Michael S., Rosenthal Patrick, Segata Nicola, Shaffer Michael, Shiffer Arron, Sinha Rashmi, Song Se Jin, Spear John R., Swafford Austin D., Thompson Luke R., Torres Pedro J., Trinh Pauline, Tripathi Anupriya, Turnbaugh Peter J., Ul-Hasan Sabah, der Hooft Justin J. J. van, Vargas Fernando, Vázquez-Baeza Yoshiki, Vogtmann Emily, Hippel Max von, Walters William, Wan Yunhu, Wang Mingxun, Warren Jonathan, Weber Kyle C., Williamson Charles H. D., Willis Amy D., Xu Zhenjiang Zech, Zaneveld Jesse R., Zhang Yilong, Zhu Qiyun, Knight Rob, Caporaso J. Gregory (2019). Reproducible, interactive, scalable and extensible microbiome data science using QIIME 2. Nature Biotechnology.

[B6423962] Callahan Benjamin J, McMurdie Paul J, Rosen Michael J, Han Andrew W, Johnson Amy Jo A, Holmes Susan P (2016). DADA2: High-resolution sample inference from Illumina amplicon data. Nature Methods.

[B6423973] Capuzzo Caterina, Firrao Giuseppe, Mazzon Luca, Squartini Andrea, Girolami Vincenzo (2005). ‘*Candidatus* Erwinia dacicola’, a coevolved symbiotic bacterium of the olive fly *Bactrocera
oleae* (Gmelin). International Journal of Systematic and Evolutionary Microbiology.

[B6424202] Caspi R., Foerster H., Fulcher C. A., Hopkinson R., Ingraham J., Kaipa P., Krummenacker M., Paley S., Pick J., Rhee S. Y., Tissier C., Zhang P., Karp P. D. (2006). MetaCyc: a multiorganism database of metabolic pathways and enzymes. Nucleic Acids Research.

[B7029572] Chen Bosheng, Teh Beng-Soon, Sun Chao, Hu Sirui, Lu Xingmeng, Boland Wilhelm, Shao Yongqi (2016). Biodiversity and Activity of the Gut Microbiota across the Life History of the Insect Herbivore Spodoptera littoralis. Scientific Reports.

[B6454582] Chen Bosheng, Du Kaiqian, Sun Chao, Vimalanathan Arunprasanna, Liang Xili, Li Yong, Wang Baohong, Lu Xingmeng, Li Lanjuan, Shao Yongqi (2018). Gut bacterial and fungal communities of the domesticated silkworm (*Bombyx
mori*) and wild mulberry-feeding relatives. The ISME Journal.

[B6424233] Chen Bosheng, Xie Sen, Zhang Xiancui, Zhang Nan, Feng Huihui, Sun Chao, Lua Xingmeng, Shao Yongqi (2020). Gut microbiota metabolic potential correlates with body size between mulberry‐feeding lepidopteran pest species. Pest Management Science.

[B6424246] Chen S. H., Yu P. Y., Sun C. H., T'an C. H., Zia Y. (1986). Fauna Sinica (Insecta: Coleoptera: Hispidae).

[B6424726] Colman D. R., Toolson E. C., Takacs-Vesbach C. D. (2012). Do diet and taxonomy influence insect gut bacterial communities?. Molecular Ecology.

[B6904327] Dillon R J, Dillon V M (2004). The gut bacteria of insects: nonpathogenic interactions.. Annual Review of Entomology.

[B6424283] Dillon R. J., Webster G., Weightman A. J., Keith Charnley A. (2010). Diversity of gut microbiota increases with aging and starvation in the desert locust. Antonie Van Leeuwenhoek.

[B6423553] Douglas Angela E. (2015). Multiorganismal insects: Diversity and function of resident microorganisms. Annual Review of Entomology.

[B6424301] Douglas GM, Maffei VJ, Zaneveld J, Yurgel SN, Langille MGI (2020). PICRUSt2: An improved and customizable approach for metagenome inference. Biorxiv.

[B6904399] Dumonteil Eric, Ramirez-Sierra Maria-Jesus, Pérez-Carrillo Silvia, Teh-Poot Christian, Herrera Claudia, Gourbière Sébastien, Waleckx Etienne (2018). Detailed ecological associations of triatomines revealed by metabarcoding and next-generation sequencing: implications for triatomine behavior and Trypanosoma cruzi transmission cycles. Scientific Reports.

[B6904248] Engel P., Martinson V. G., Moran N. A. (2012). Functional diversity within the simple gut microbiota of the honey bee. Proceedings of the National Academy of Sciences.

[B6904257] Engel Philipp, Moran Nancy A. (2013). Functional and evolutionary insights into the simple yet specific gut microbiota of the honey bee from metagenomic analysis. Gut Microbes.

[B6424320] Engel P., Moran N. A. (2013). The gut microbiota of insects - diversity in structure and function. FEMS Microbiol Reviews.

[B6424329] Fegatella F., Cavicchioli R. (2000). Physiological responses to starvation in the marine oligotrophic ultramicrobacterium *Sphingomonas* sp. strain RB2256. Applied and Environmental Microbiology.

[B7029584] Ferguson Laura V., Dhakal Pranav, Lebenzon Jacqueline E., Heinrichs David E., Bucking Carol, Sinclair Brent J. (2018). Seasonal shifts in the insect gut microbiome are concurrent with changes in cold tolerance and immunity. Functional Ecology.

[B7023716] Gong Beini, Wu Pingxiao, Huang Zhujian, Li Yuewu, Dang Zhi, Ruan Bo, Kang Chunxi, Zhu Nengwu (2016). Enhanced degradation of phenol by Sphingomonas sp. GY2B with resistance towards suboptimal environment through adsorption on kaolinite.. Chemosphere.

[B6424338] Gouveia C., Asensi M. D., Zahner V., Rangel E. F., Oliveira S. M. (2008). Study on the bacterial midgut microbiota associated to different Brazilian populations of *Lutzomyia
longipalpis* (Lutz & Neiva) (Diptera: Psychodidae). Neotropical Entomology.

[B6424357] He Z., Yin Y., Cao Y., Dong Y., Zhang W. (2001). Study on the *Apriona
germari* (Hope) larvae's intestinal bacterial flora. Acta Microbiologica Sinica.

[B6424367] Hooper L. V., Gordon J. I. (2001). Commensal host-bacterial relationships in the gut. Science.

[B6424376] Huang S., Zhang H. (2017). The impact of environmental heterogeneity and life stage on the hindgut microbiota of *Holotrichia
parallela* larvae (Coleoptera: Scarabaeidae). PLOS ONE.

[B7023771] Innerebner Gerd, Knief Claudia, Vorholt Julia A. (2011). Protection of Arabidopsis thaliana against Leaf-Pathogenic Pseudomonas syringae by Sphingomonas Strains in a Controlled Model System. Applied and Environmental Microbiology.

[B6424385] Kanehisa Minoru, Goto Susumu (2000). KEGG: Kyoto encyclopedia of genes and genomes. Nucleic Acids Research.

[B6424394] Karamipour N., Fathipour Y., Mehrabadi M. (2016). Gammaproteobacteria as essential primary symbionts in the striped shield bug, *Graphosoma
lineatum* (Hemiptera: Pentatomidae). Scientific Reports.

[B6424403] Kashkouli M., Fathipour Y., Mehrabadi M. (2019). Heritable Gammaproteobacterial symbiont improves the fitness of *Brachynema
germari* Kolenati (Hemiptera: Pentatomidae). Environmental Entomology.

[B6424421] Kramer P., Bressan P. (2015). Humans as superorganisms: How microbes, viruses, imprinted genes, and other selfish entities shape our behavior. Perspectives on Psychological Science.

[B6424430] Kuzina L. V., Peloquin J. J., Vacek D. C., Miller T. A. (2001). Isolation and identification of bacteria associated with adult laboratory Mexican fruit flies, *Anastrepha
ludens* (Diptera: Tephritidae). Current Microbiology.

[B6424439] Laskin A. I., White D. C. (1999). Preface to special issue on *Sphingomonas*. Journal of Industrial Microbiology and Biotechnology.

[B6424457] Liu Y., Shen Z., Yu J., Li Z., Liu X., Xu H. (2020). Comparison of gut bacterial communities and their associations with host diets in four fruit borers. Pest Management Science.

[B6424468] Louca S., Doebeli M. (2018). Efficient comparative phylogenetics on large trees. Bioinformatics.

[B6424477] Martin M (2011). Cutadapt removes adapter sequences from high-throughput sequencing reads. EMBnet.Journal.

[B6903278] Michell Craig T., Nyman Tommi (2021). Microbiomes of willow-galling sawflies: effects of host plant, gall type, and phylogeny on community structure and function. Genome.

[B6424486] Mizrahi-Man O, Davenport ER, Gilad Y (2013). Taxonomic classification of bacterial 16S rRNA genes using short sequencing reads: Evaluation of effective study designs. PLOS ONE.

[B6424495] Nicholas AB, Benjamin DK, Jai RR, Matthew D, Evan B, Rob K, Gavin AH, Caporaso JG (2018). Optimizing taxonomic classification of marker-gene amplicon sequences with qiime 2’s q2-feature-classifier plugin. Microbiome.

[B7006677] Oh San Na, Seo Mi Ja, Youn Young Nam, Yu Yong Man (2015). Antifungfal Activity Against Plant Pathogenic Fungi on Insect Enterobacteriaceae. The Korean Journal of Pesticide Science.

[B7003765] Ondov Brian D, Bergman Nicholas H, Phillippy Adam M (2011). Interactive metagenomic visualization in a Web browser. BMC Bioinformatics.

[B6424508] Prabhakar C. S., Sood P., Kanwar S. S., Sharma P. N., Kumar A., Mehta P. K. (2013). Isolation and characterization of gut bacteria of fruit fly, *Bactrocera
tau* (Walker). Phytoparasitica.

[B6424519] Price M. N., Dehal P. S., Arkin A. P. (2010). FastTree 2--approximately maximum-likelihood trees for large alignments. PLOS ONE.

[B6897451] Quast Christian, Pruesse Elmar, Yilmaz Pelin, Gerken Jan, Schweer Timmy, Yarza Pablo, Peplies Jörg, Glöckner Frank Oliver (2012). The SILVA ribosomal RNA gene database project: improved data processing and web-based tools. Nucleic Acids Research.

[B7029343] Rajan Resma, Chunduri Alekhya Rani, Lima Anugata, Mamillapalli Anitha (2020). 16S rRNA sequence data of Bombyx
mori gut bacteriome after spermidine supplementation.. BMC Research Notes.

[B6904811] Russell Jacob A, Moreau Corrie S, Goldman-Huertas Benjamin, Fujiwara Mikiko, Lohman David J, Pierce Naomi E (2009). Bacterial gut symbionts are tightly linked with the evolution of herbivory in ants.. Proceedings of the National Academy of Sciences of the United States of America.

[B6904336] Sabree Zakee L, Moran Nancy A (2014). Host-specific assemblages typify gut microbial communities of related insect species. SpringerPlus.

[B6424542] Salvucci E. (2019). The human-microbiome superorganism and its modulation to restore health. International Journal of Food Sciences and Nutrition.

[B6424551] Schloss PD, Delalibera I, Handelsman J, Raffa KF (2006). Bacteria associated with the guts of two wood-boring beetles: *Anoplophora
glabripennis* and *Saperda
vestita* (Cerambycidae). Environmental Entomology.

[B7029293] Shinde Ashok A., Shaikh Faiyaz K., Gadge Prafull P., Padul Manohar V., Govindwar Sanjay P., Kachole Manvendra S. (2019). Conserved nature of Helicoverpa armigera gut bacterial flora on different host plants and in vitro interactions with PI proteins advocates role in host digestive physiology. Journal of the Saudi Society of Agricultural Sciences.

[B6424560] Shi Weibing, Syrenne Ryan, Sun Jian-Zhong, Yuan Joshua S. (2010). Molecular approaches to study the insect gut symbiotic microbiota at the ‘omics’ age. Insect Science.

[B6424569] Silva C., Cabral J. M., van Keulen F. (2004). Isolation of a beta-carotene over-producing soil bacterium, *Sphingomonas* sp. Biotechnology Letters.

[B6424578] Skidmore I. H., Hansen A. K. (2017). The evolutionary development of plant-feeding insects and their nutritional endosymbionts. Insect Science.

[B6424587] Sleator R. D. (2010). The human superorganism - of microbes and men. Medical Hypotheses.

[B7029595] Tagliavia Marcello, Messina Enzo, Manachini Barbara, Cappello Simone, Quatrini Paola (2014). The gut microbiota of larvae of Rhynchophorus ferrugineus Oliver (Coleoptera: Curculionidae). BMC Microbiology.

[B6903057] Vilanova Cristina, Baixeras Joaquín, Latorre Amparo, Porcar Manuel (2016). The Generalist Inside the Specialist: Gut Bacterial Communities of Two Insect Species Feeding on Toxic Plants Are Dominated by Enterococcus sp.. Frontiers in Microbiology.

[B6424596] Wang H., Zhang J-Y., Wang X-M., Hu H-L., Xia R-X., Li Q., Zhu X-W., Wang T-M., Liu Y-Q., Qin L. (2020). Comparison of bacterial communities between midgut and midgut contents in two silkworms, *Antheraea
pernyi* and *Bombyx
mori*. Scientific Reports.

[B7023729] Wang Xia, Xu Ping, Yuan Yong, Liu Changlong, Zhang Dezhong, Yang Zhengting, Yang Chunyu, Ma Cuiqing (2006). Modeling for Gellan Gum Production by Sphingomonas paucimobilis ATCC 31461 in a Simplified Medium. Applied and Environmental Microbiology.

[B6424611] Wang Y., Gilbreath T. M., Kukutla P., Yan G., Xu J. (2011). Dynamic gut microbiome across life history of the malaria mosquito *Anopheles
gambiae* in Kenya. PLOS ONE.

[B7029606] Wang Zhibo, Li Hong, Zhou Xiaogui, Tang Meijun, Sun Liang, Zhan Shuai, Xiao Qiang (2020). Comparative characterization of microbiota between the sibling species of tea geometrid moth *Ectropis
obliqua* Prout and E. grisescens Warren. Bulletin of Entomological Research.

[B6424621] Warnecke Falk, Luginbühl Peter, Ivanova Natalia, Ghassemian Majid, Richardson Toby H., Stege Justin T., Cayouette Michelle, McHardy Alice C., Djordjevic Gordana, Aboushad Nahla (2007). Metagenomic and functional analysis of hindgut microbiota of a wood-feeding higher termite. Nature.

[B6904310] Wu Dongying, Daugherty Sean C, Van Aken Susan E, Pai Grace H, Watkins Kisha L, Khouri Hoda, Tallon Luke J, Zaborsky Jennifer M, Dunbar Helen E, Tran Phat L, Moran Nancy A, Eisen Jonathan A (2006). Metabolic Complementarity and Genomics of the Dual Bacterial Symbiosis of Sharpshooters. PLoS Biology.

[B6904796] Xia Xiaofeng, Zheng Dandan, Zhong Huanzi, Qin Bingcai, Gurr Geoff M., Vasseur Liette, Lin Hailan, Bai Jianlin, He Weiyi, You Minsheng (2013). DNA Sequencing Reveals the Midgut Microbiota of Diamondback Moth, Plutella
xylostella (L.) and a Possible Relationship with Insecticide Resistance. PLoS ONE.

[B6424735] Xia Xiaofeng, Gurr Geoff M., Vasseur Liette, Zheng Dandan, Zhong Huanzi, Qin Bingcai, Lin Junhan, Wang Yue, Song FengQin, Li Yong, Lin Hailan, You Minsheng (2017). Metagenomicsequencing of diamondback moth gut microbiome unveils key holobiont adaptations for herbivory. Frontiers in Microbiology.

[B6904689] Xia Xiaofeng, Lan Bomiao, Tao Xinping, Lin Junhan, You Minsheng (2020). Characterization of Spodoptera
litura Gut Bacteria and Their Role in Feeding and Growth of the Host. Frontiers in Microbiology.

[B6424668] Xu Z., Xia M., Huo Y. X., Yang Y. (2020). *Intestinirhabdus
alba* gen. nov., sp. nov., a novel genus of the family Enterobacteriaceae, isolated from the gut of plastic-eating larvae of the Coleoptera insect *Zophobas
atratus*. International Journal of Systematic and Evolutionary Microbiology.

[B6424677] Ye Y., Doak T. G. (2009). A parsimony approach to biological pathway reconstruction/inference for genomes and metagenomes. PLOS Computational Biology.

[B6424686] Yun J. H., Roh S. W., Whon T. W., Jung M. J., Kim M. S., Park D. S., Yoon C., Nam Y. D., Kim Y. J., Choi J. H., Kim J. Y., Shin N. R., Kim S. H., Lee W. J., Bae J. W. (2014). Insect gut bacterial diversity determined by environmental habitat, diet, developmental stage, and phylogeny of host. Applied and Environmental Microbiology.

[B7029304] Zeng JianYong, Shi ZhongBin, Shi JianHong, Guo JiaXing, Zhang GuoCai, Zhang Jie (2019). Ambient temperature-mediated enzymic activities and intestinal microflora in Lymantria
dispar larvae.. Archives of Insect Biochemistry and Physiology.

[B6424706] Zhang Jing, Zhang Yibo, Xue Yantao, Liu Huai, Zhang Guifen, Wan Fanghao (2017). Research advances on a secondary endosymbiont *Rickettsia* in insect. Journal of Environmental Entomology.

[B7023751] Zhao Xin, Chen Zonggang, Gu Guofeng, Guo Zhongwu (2016). Recent advances in the research of bacterial glucuronosyltransferases. Journal of Carbohydrate Chemistry.

